# Fast computation of the eigensystem of genomic similarity matrices

**DOI:** 10.1186/s12859-024-05650-8

**Published:** 2024-01-25

**Authors:** Georg Hahn, Sharon M. Lutz, Julian Hecker, Dmitry Prokopenko, Michael H. Cho, Edwin K. Silverman, Scott T. Weiss, Christoph Lange

**Affiliations:** 1 T.H. Chan School of Public Health, Harvard University, Boston, MA 02115 USA; 2 Channing Divsion of Network Medicine, Brigham and Women’s Hospital, Boston, MA 02115 USA; 3 Massachusetts General Hospital, Harvard University, Boston, MA 02114 USA

**Keywords:** Covariance matrix, Fast SVD, Genomic relationship matrix, Jaccard matrix, Principal components, Weighted Jaccard matrix

## Abstract

The computation of a similarity measure for genomic data is a standard tool in computational genetics. The principal components of such matrices are routinely used to correct for biases due to confounding by population stratification, for instance in linear regressions. However, the calculation of both a similarity matrix and its singular value decomposition (SVD) are computationally intensive. The contribution of this article is threefold. First, we demonstrate that the calculation of three matrices (called the covariance matrix, the weighted Jaccard matrix, and the genomic relationship matrix) can be reformulated in a unified way which allows for the application of a randomized SVD algorithm, which is faster than the traditional computation. The fast SVD algorithm we present is adapted from an existing randomized SVD algorithm and ensures that all computations are carried out in sparse matrix algebra. The algorithm only assumes that row-wise and column-wise subtraction and multiplication of a vector with a sparse matrix is available, an operation that is efficiently implemented in common sparse matrix packages. An exception is the so-called Jaccard matrix, which does not have a structure applicable for the fast SVD algorithm. Second, an approximate Jaccard matrix is introduced to which the fast SVD computation is applicable. Third, we establish guaranteed theoretical bounds on the accuracy (in $L_2$ norm and angle) between the principal components of the Jaccard matrix and the ones of our proposed approximation, thus putting the proposed Jaccard approximation on a solid mathematical foundation, and derive the theoretical runtime of our algorithm. We illustrate that the approximation error is low in practice and empirically verify the theoretical runtime scalings on both simulated data and data of the 1000 Genome Project.

## Introduction

In computational genomics, the computation of eigenvectors as part of a principal component analysis (PCA) is a widespread method to infer population structure and to correct for confounding due to ancestry. It has long been known that case–control studies are subject to population stratification which can induce significant spurious associations at loci that are unrelated with a response [22]. For instance, in [3] the authors highlight such a spurious association through stratification by showing that a SNP in the lactase gene LCT varies widely in frequency across Europe and was strongly associated with height. To correct for stratification in genome-wide association studies [17], methodology such as EIGENSTRAT [18] has been subsequently developed. Further computational improvements are available [16]. Further works in the literature address confounding induced by population stratification with the help of a two-step procedure [5], or PCA analyses with tens of thousands of single-nucleotide polymorphisms (SNPs) to infer population structure [14].

This article focuses on the fast computation of eigenvectors of four different similarity matrices. These matrices provide pairwise similarity measures between genomes, and their eigenvectors are popular means to correct for population stratification. We consider the (classic) covariance matrix [24], the Jaccard matrix [13, 19], the weighted Jaccard matrix [25], and the genomic relationship matrix (GRM) [30]. All four matrices are computed on genomic input $G \in \mathbb {R}^{n \times m}$, where $n \in \mathbb {N}$ is the number of loci and $m \in \mathbb {N}$ is the number of individuals. The matrix *G* is usually sparse. All four similarity matrices have dimensions $m \times m$, where each entry (*i*, *j*) is a similarity measure between the genomic data of individuals *i* and *j*. All four matrices are symmetric by definition.

In real data applications such as the 1000 Genomes Project [28] or the UK Biobank [27], the number of individuals quickly reaches numbers in the thousands, ten thousands, or hundred thousands. In this case, traditional eigenvector computations, for instance using the function *eigen* in R [21], become infeasible. Alternative methods with lower computational complexity are iterative methods such as the *power method* [29], also called Von Mises iteration, or the truncated singular value decomposition (SVD) implemented in, for instance, the R-package *RSpectra* [20]. However, for these alternatives to be applicable, the complete similarity matrix of dimension $m \times m$ has to be computed first before extracting its eigenvectors. In the best case, calculating a similarity matrix is computationally intensive itself, while in the worst case, its calculation is computationally infeasible. The latter case occurs since the similarly matrices are usually dense even when computed on sparse genomic input *G*.

As demonstrated in the literature [12], for a real-valued matrix *X* the eigenvectors of the matrices $X^\top X$ and $X X^\top$ can be computed without actually calculating $X^\top X$ or $X X^\top$. This is advantageous in genomic applications, since $X^\top X$ and $X X^\top$ are typically dense even for sparse *X*, and can thus be infeasible to compute. Since the algorithm of [12] only works for eigenvectors of matrices which can be expressed as the product $X^\top X$ or $X X^\top$, the task is to provide decompositions of the four similarity matrices we consider in the form $X^\top X$ or $X X^\top$.

A couple of remarks are in order on the two levels of approximations that are considered in this article. First, the randomized SVD algorithm of [12] does not compute the (numerically) exact eigenvectors of $X^\top X$ or $X X^\top$ for a given input *X*, but an approximation thereof. Second, the randomized SVD algorithm assumes that its input is of the form $X^\top X$ or $X X^\top$. As shown in the article, this applies to three similarity measures (the covariance matrix, the weighted Jaccard matrix, and the genomic relationship matrix), but not to the Jaccard matrix. Therefore, we approximate the Jaccard matrix itself with a surrogate of form $X^\top X$ in order to apply the randomized SVD algorithm.

The contribution of this article is threefold. First, we show that the eigenvectors of the covariance matrix, the weighted Jaccard matrix, and the genomic relationship matrix can be computed efficiently using the randomized SVD algorithm by rewriting their computations in a unified way in the form $X^\top X$ or $X X^\top$. To this end, we propose a tailored algorithm by adapting the randomized SVD algorithm of [12]. The tailored algorithm never actually computes any of the similarity matrices and fully supports sparse matrix algebra for efficient calculations. The tailored algorithm only assumes that row-wise and column-wise subtraction and multiplication of a vector with a sparse matrix is implemented efficiently. Second, we propose an approximate Jaccard matrix which likewise allows for an efficient computation of its eigenvectors via fast SVD without actually computing the similarity measure. Third, we establish guaranteed theoretical bounds on the distance (in $L_2$ norm and angle) between the eigenvectors of the Jaccard matrix and the ones of our proposed approximation, thus putting the proposed Jaccard approximation on a solid mathematical foundation. Moreover, we derive the theoretical runtime of the fast SVD computation for all four approaches.

In an experimental section, we illustrate the exactness of the proposed computations for the covariance matrix, the weighted Jaccard matrix, and the genomic relationship matrix. Moreover, we experimentally verify the theoretical runtime derivations, showing that indeed, the fast SVD computation outperforms the traditional SVD computation. Special attention is given to the Jaccard matrix. Using simulated data, we verify the proven theoretical bounds on the distance between the eigenvectors of the Jaccard matrix and our proposed approximation, showing that the approximation error is very low in practice. Moreover, we demonstrate the (visual) trade-off in accuracy between the Jaccard matrix and its approximation for population stratification plots using data of the 1000 Genomes Project.

The computation of a randomized PCA has also been considered in [1]. In their publication, the authors likewise adapt the original algorithm of [12]. However, the authors only consider the genomic relationship matrix, they do not present computations for fully sparse matrix algebra, and they do not establish a unified framework allowing one to extend the fast PCA computation to the other similarity matrices as well. Importantly, the Jaccard approximation and the theoretical bounds on the accuracy of the approximation we prove are unconsidered.

The paper is structured as follows. Section “Methods” introduces the proposed decomposition of the four similarity matrices under consideration (section “Fast computation of eigenvectors”), establishes that the fast computation of eigenvectors applies to three of these matrices (section “Decomposition of three similarity matrices”), introduces a new approximation of the Jaccard similarity matrix (section “A new approximation of the Jaccard similarity matrix”), establishes theoretical bounds on the accuracy of the approximation (section “Theoretical error bounds on the eigenvectors of the Jaccard approximation”), and summarizes all findings as an efficient algorithm (section “An efficient algorithm using sparse matrix algebra”) together with asymptotic runtime considerations (section “Runtime considerations”). All experimental results can be found in section “Experimental results”. The article concludes with a discussion in section “Discussion”.

The proposed methodology has been implemented as part of the R-package *locStra* [8, 11], available on the Comprehensive R Archive Network [21]. In the entire article, $\mathbb {0}_n$ and $\mathbbm {1}_n$ denote the column vectors of length *n* with all entries set to 0 or 1, respectively. Moreover, we denote with $\sigma _r(Y) \in \mathbb {R}^n$ and $\sigma _c(Y) \in \mathbb {R}^m$ the row and column sums of a matrix $Y \in \mathbb {R}^{n \times m}$, and with $\mu _r(Y) \in \mathbb {R}^n$ and $\mu _c(Y) \in \mathbb {R}^m$ the row and column means of *Y*, respectively. As usual, the notation $v \otimes w$ is used to denote the outer product between two vectors *v* and *w*, and ${{\,\textrm{diag}\,}}(v)$ denotes the square matrix having zero entries except from vector *v* on its diagonal.

## Methods

This section demonstrates that the covariance matrix, the weighted Jaccard matrix, and the genomic relationship matrix can be expressed in a unified way which allows for an efficient computation of their eigenvectors (sections “Fast computation of eigenvectors” and “Decomposition of three similarity matrices”). This does not apply to the Jaccard matrix, for which we propose a new approximation instead that allows for a fast eigenvector computation (section “A new approximation of the Jaccard similarity matrix”). Importantly, we establish theoretical bounds on the accuracy of the eigenvectors obtained from the Jaccard approximation (section “Theoretical error bounds on the eigenvectors of the Jaccard approximation”). We summarize all findings in an algorithm tailored to the four similarity matrices in section “An efficient algorithm using sparse matrix algebra”. We conclude with considerations on the asymptotic speedup in section “Runtime considerations”.

### Fast computation of eigenvectors

The algorithm of [12] allows one to compute the eigenvectors of either the matrix $X^\top X$, or the matrix $X X^\top$ by considering $X \in \mathbb {R}^{n \times m}$ only, where $n,m \in \mathbb {N}$. The actual matrix product $X^\top X$ or $X X^\top$ does not need to be computed at any point in time. This is advantageous if the matrix *X* is sparse since then, oftentimes, $X^\top X$ and $X X^\top$ are dense. In the following, we focus on the computation of the eigenvectors of $X^\top X$ only.

The idea of the randomized SVD of [12] can be summarized as follows. Given a matrix *X*, the aim of the randomized SVD is to compute a low-rank matrix approximation $X=U \Sigma V^\top$. To this end, we first compute an approximate basis for the range of *X*, that is an orthonormal matrix *Q* such that $X \approx Q Q^\top X$ (effectively, this is a low-rank matrix factorization $X \approx AB$ with $A \in \mathbb {R}^{n \times r}$, $B \in \mathbb {R}^{r \times m}$ and $r \ll n,m$). The dimension of *Q* can be chosen by the user and controls the accuracy of the approximation. Setting $B = Q^\top X$, the efficiency of the randomized SVD comes from *B* being much smaller than *X*. After computing the SVD of *B* as $B = \tilde{U} \Sigma V^\top$, one obtains $X \approx Q B = Q \tilde{U} \Sigma V^\top$. Therefore, setting $U = Q \tilde{U}$ results in a low-rank approximation $X \approx U \Sigma V^\top$.

In order to apply the fast eigenvector computation of [12], we need to express all similarity matrices under consideration as a product of the form $X^\top X$. This is not a straightforward task, as the computation of the aforementioned similarity matrices involves normalization and centering operations. As an additional complication, the normalization and centering operations usually destroy the sparseness of *X*. Therefore, these operations are kept separate in the following formulas. We consider matrices *X* which can be expressed as

1 $$\begin{aligned} X&= a \cdot (G - \mathbbm {1}_n \otimes w_1) {{\,\textrm{diag}\,}}(v_1) \end{aligned}$$

or alternatively, as

2 $$\begin{aligned} X&= a \cdot {{\,\textrm{diag}\,}}(v_2) (G - w_2 \otimes \mathbbm {1}_m), \end{aligned}$$

where $G \in \mathbb {R}^{n \times m}$ is the genomic input data (where $n \in \mathbb {N}$ is the number of loci and $m \in \mathbb {N}$ is the number of individuals), $a \in \mathbb {R}$ is a scalar, and $v_1, w_1 \in \mathbb {R}^m$ as well as $v_2, w_2 \in \mathbb {R}^n$ are vectors of appropriate dimensions. The scalar *a* is kept separate and not absorbed into *v* for clarity of notation, as most similiarity matrices have a separate normalizing constant. The notation $(\cdot )$ denotes the multiplication of a scalar with a matrix.

The expressions in Eqs. (1) and (2) are not suitable for actual computations since *G* is assumed sparse, while the matrices $\mathbbm {1}_n \otimes w_1$ (encoding the subtraction of $w_1$ from all rows of *G*) and $w_2 \otimes \mathbbm {1}_m$ (encoding the subtraction of $w_2$ from all columns of *G*) are dense. Instead, we assume that there are efficient row-wise and column-wise subtraction and multiplication operations available which operate directly on *G* in sparse matrix algebra. Such operations are routinely available in sparse matrix packages such as the *Matrix* package in R [2].

We denote with $\odot _r$, $\ominus _r$ as well as $\odot _c$, $\ominus _c$ the row/column-wise multiplication and subtraction operation of a vector with a (sparse) matrix, respectively. To be precise, $G \ominus _r w$ subtracts *w* from all rows of *G*, and $G \ominus _c w$ subtracts *w* from all columns of *G*. Analogously, $v \odot _r G$ multiplies all rows of *G* with *v*, and $v \odot _c G$ multiplies all columns of *G* with *v* (assuming *v* and *w* are of appropriate dimensions). Using these operations, we can express Eq. (1) as

3 $$\begin{aligned} X = a \cdot v_1 \odot _r (G \ominus _r w_1), \end{aligned}$$

and Eq. (2) becomes

4 $$\begin{aligned} X = a \cdot v_2 \odot _c (G \ominus _c w_2). \end{aligned}$$

As shown in the following sections (sections “Decomposition of three similarity matrices” and “A new approximation of the Jaccard similarity matrix”), the covariance matrix, the weighted Jaccard matrix, the genomic relationship matrix, and a newly proposed Jaccard approximation can be expressed in a unified form as $X^\top X$, with *X* as in Eq. (3) and Eq. (4).

While *G* is usually a sparse matrix, centering *G* with a vector *w* as done in Eqs. (3) and (4) usually results in a dense matrix, which is computationally inefficient to handle or even infeasible. As shown in section “An efficient algorithm using sparse matrix algebra”, the main advantage of Eqs. (3) and (4) consists in the fact that they allow one to compute eigenvectors in sparse algebra only, without ever performing the multiplication or subtraction operations.

### Decomposition of three similarity matrices

The covariance matrix, the weighted Jaccard matrix, and the genomic relationship matrix allow for an expression of the form of Eqs. (3) or (4):

1. The covariance matrix is computed as $\frac{1}{n-1} G^\top G$ after centering all rows of *G* with their respective column means. This fits into the framework of Eq. (3) by setting $a = \frac{1}{\sqrt{n-1}}$, $v_1 = \mathbbm {1}_m \in \mathbb {R}^m$, and $w_1 = \mu _c(G) \in \mathbb {R}^m$.
2. The computation of the weighted Jaccard matrix [25] is more involved and repeated here for convenience. First, a quantity *numAlleles* is computed as 2*n*. Then, the sum of variants in *G* is computed as the row sums of *G* and denoted as *sumVariants*. In a pre-processing step to invert the minor alleles, all rows in *G* are inverted if their sum of variants is strictly larger than *n*. Second, a set of weights is computed as follows. A quantity *totalPairs* is computed as $$s(s-1)/2$$, where $$s \in \mathbb {R}^n$$ is the vector of row sums of *G* and the vector multiplication is performed componentwise. The weight vector *weights* is then computed as *numAlleles*$$*($$*numAlleles*$$-1)/$$*totalPairs*, again taking all operations to be componentwise. The vectors *totalPairs* and *weights* both have dimension *n*. Third, the weighted Jaccard matrix is computed as $$\frac{1}{4n}$$ $$(G \odot _c weights)^\top G$$. This computation fits into the framework of Eq. (4) by setting $$a = \frac{1}{\sqrt{4n}}$$, $$v_2 = \sqrt{weights}$$ (with the square root operation performed componentwise), and $$w_2 = \mathbb {0}_n \in \mathbb {R}^n$$.
3. The genomic relationship matrix [30] exists in two flavors, a robust and a non-robust version. Both are easily defined as follows. Define $p = \mu _r(G)/2$ as row means of *G*, and $q = 2p(1-p)$, where the vector multiplication is again understood componentwise. Let *s* be the sum of all entries in *q*. After centering the columns of *G* with 2*p* (that is, $X \leftarrow X \ominus _c (2p)$), the robust GRM is defined as $G^\top G /s$, and the non-robust GRM is defined as $\frac{1}{n} G^\top (G \odot _c q^{-1})$, where the inverse operation $q^{-1}$ is understood componentwise. Both the robust and non-robust versions of the GRM fit into the framework of Eq. (4). For the robust GRM, we set $a = \frac{1}{\sqrt{s}}$, $v_2 = \mathbbm {1}_n \in \mathbb {R}^n$, and $w_2 = 2p$. For the non-robust GRM, we set $a = \frac{1}{\sqrt{n}}$, $v_2 = \frac{1}{\sqrt{q}}$, again taking all vector operations to be componentwise, and $w_2 = 2p$.

Finally, it remains to note that the Jaccard matrix [19] does not allow for a decomposition into $X^\top X$ with an appropriately chosen matrix *X*. This is easily verified in practice. Indeed, it is not complicated to find a simulated or real life genomic dataset for which the Jaccard matrix has negative eigenvalues, thus making it not positive (semi-)definite. This proves that a decomposition into the form $X^\top X$, which necessarily implies positive (semi-)definiteness, is impossible.

### A new approximation of the Jaccard similarity matrix

Since the fast SVD computation of [12] is not applicable to the Jaccard similarity matrix [19], applications in genomics which rely on the Jaccard matrix either in the form of population stratification plots [10, 15] or to correct genome-wide association studies [9], are severely limited from a computational standpoint. In order to be able to scale such computations, a modification of the Jaccard matrix is required that enables the fast SVD computation of [12]. Any such modification necessarily results in an approximation of the original Jaccard matrix, though the error of the proposed approximation will be quantified in section “Theoretical error bounds on the eigenvectors of the Jaccard approximation”. As a side effect, the proposed approximation of the Jaccard matrix will be positive definite (in fact, all matrices *X* for which the eigenvectors of $X^\top X$ are computed with the help of the randomized SVD algorithm must have this property), which is desirable from the standpoint of numerical stability.

The Jaccard matrix is computed as follows on a *binary* genomic input matrix *G*. First, a matrix $A \in \mathbb {R}^{n \times n}$ is computed. Each entry (*i*, *j*) in *A* is obtained by computing the logical *and* operation on the binary columns *i* and *j* of *G*, and storing the sum of ones (or values *True*) in the resulting vector in $A_{i,j}$. Similarly, a matrix $O \in \mathbb {R}^{n \times n}$ is computed whose entry (*i*, *j*) represents the sum of ones after an *or* operation on the binary columns *i* and *j* of *G*. The Jaccard matrix *J* is then computed as $J = A/O$, where the matrix division is taken componentwise.

It is important to note that for binary matrices, the logical *and* operation required to compute $A \in \mathbb {R}^{n \times n}$ is equivalent to simply computing the matrix-matrix product of *G* with its transpose, that is $A = G^\top G$. Therefore, it is in fact the *or* operation that prevents the Jaccard matrix from being expressible in the form $X^\top X$.

To fix this, we propose a simple approximation that replaces the computation of the matrix *O*. Note that, when computing the logical *or* operation on two columns *i* and *j* of *G*, the maximal number of ones we can obtain in the resulting vector is $2 \max (s)$, where *s* is the vector of column sums of *G*. This case occurs if both columns of *G* contain the maximal number of nonzero entries, though at different positions each. To be conservative, we therefore propose to compute an approximation of $J = A/O$ as $\hat{J} = \frac{1}{2\max (s)} A$. The matrix $\hat{J}$ has the property that any entry in $\hat{J}$ is at most as large as the corresponding one in *J*, thus never overestimating the similarity between two individuals.

Due to its simpler structure, the approximate Jaccard matrix $\hat{J} = \frac{1}{2\max (s)} A$ fits into the framework of Eq. (3) by taking $a = \frac{1}{\sqrt{2\max (s)}}$, $v_1 = \mathbbm {1}_m \in \mathbb {R}^m$, and $w_1 = \mathbb {0}_m \in \mathbb {R}^m$, where *s* was the vector of column sums of $G \in \mathbb {R}^{n \times m}$.

### Theoretical error bounds on the eigenvectors of the Jaccard approximation

The original Jaccard matrix as defined in [19] and the proposed approximate Jaccard matrix of section “A new approximation of the Jaccard similarity matrix” naturally differ slightly, and so do their eigenvectors.

Therefore, our proposed approximate Jaccard matrix comes with a classical speed/ accuracy tradeoff. Its eigenvectors are much faster to compute, though at the expense of a (slight) loss in accuracy. However, as the computation of eigenvectors is at the heart of this paper, this section establishes guaranteed theoretical bounds on the distance (in $L_2$ norm and angle) between the eigenvectors of the Jaccard matrix and the ones of our proposed approximation. These a priori bounds allow the user to gauge in advance the trade-off between obtained speedup and sacrificed accuracy.

To derive the bounds, we make use of the so-called “Davis-Kahan $\sin (\theta )$” theorem [4]. Citing the statement of the theorem in [23], let $A \in \mathbb {R}^{d \times d}$ and $\hat{A} \in \mathbb {R}^{d \times d}$ be two symmetric matrices with eigen decompostions given by $A = \sum _{i=1}^d \lambda _i u_i u_i^\top$ and $\hat{A} = \sum _{i=1}^d \hat{\lambda }_i \hat{u}_i \hat{u}_i^\top$, where $\lambda _1 \ge \cdots \ge \lambda _d$ and $\hat{\lambda }_1 \ge \cdots \ge \hat{\lambda }_d$ are the sorted eigenvalues. Then, $|\sin (\angle (u_1,\hat{u}_1))| \le 2 \frac{\Vert A-\hat{A} \Vert _\text {op}}{\max (\lambda _1-\lambda _2,\hat{\lambda }_1-\hat{\lambda }_2)}$ and $\min _{\epsilon \in \{+1,-1\}} \Vert u_1 - \epsilon \hat{u}_1 \Vert _2 \le \sqrt{2} |\sin (\angle (u_1,\hat{u}_1))|$.

The Davis-Kahan theorem allows one to bound the distance (up to multiplication with $+1$ or $-1$, since eigenvectors are only defined up to a unit) between the eigenvectors of a matrix *A* and an arbitrary perturbation $\hat{A}$ of *A* using the angle between their eigenvectors, which in turn is bounded by a quantity involving the operator norm of $A - \hat{A}$ and their first eigenvalues.

Applied to the Jaccard matrix *J* and our proposed approximation $\hat{J}$ of section “A new approximation of the Jaccard similarity matrix”, we see that the difference between the eigenvectors $u_1$ of *J* and $\hat{u}_1$ of $\hat{J}$, up to a unit $\{+1,-1\}$, can be bounded as

5 $$\begin{aligned} \min _{\epsilon \in \{+1,-1\}} \Vert u_1 - \epsilon \hat{u}_1 \Vert _2&\le \sqrt{2} |\sin (\angle (u_1,\hat{u}_1))|\end{aligned}$$

6 $$\le 2\sqrt 2 \frac{{\left\| {J - \hat{J}} \right\|_{{{\text{op}}}} }}{{\max \left( {\lambda _{1} - \lambda _{2} ,\hat{\lambda }_{1} - \hat{\lambda }_{2} } \right)}},$$

where $\lambda _1,\lambda _2$ and $\hat{\lambda }_1,\hat{\lambda }_2$ are the first two eigenvalues of *J* and $\hat{J}$, respectively. The operator norm of the two matrices in Eq. (6) is straightforward to compute (in the simulations of section “Experimental results”, we actually again bound the operator norm by the Frobenius norm, thus making use of norm equivalence in $\mathbb {R}$), and the first two eigenvalues of the two matrices can be computed efficiently using, for instance, the power method (also called Von Mises iteration) of [29].

It is important to note that the aforementioned approximation can also be used without actually computing the eigenvalues of *J* and $\hat{J}$. This is possible with the help of the Gershgorin circle theorem [6], which allows one to easily obtain lower and upper bounds on all eigenvalues of a matrix.

### An efficient algorithm using sparse matrix algebra

The decomposition of Eqs. (3) and (4) allows one to formulate an efficient algorithm to compute the eigenvectors of $X^\top X$ using sparse algebra only. Our algorithm is adapted from the one of [12], stated again for completeness in Algorithm 1, to preserve the sparseness of the input during the entire computation.

**Algorithm 1:**
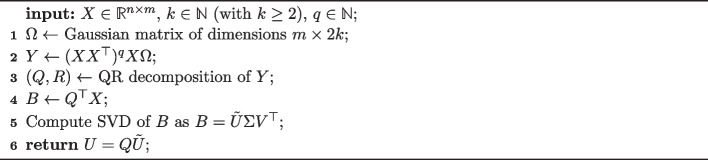
randomized fast SVD

Algorithm 1 returns the first *k* principal components of $X^\top X$ for an input matrix $X \in \mathbb {R}^{n \times m}$. A full SVD can also be obtained as $U \Sigma V^\top$, where *U* is computed in step 6, and $\Sigma$ and $V^\top$ come from the SVD of matrix *B* in step 5. The parameter *q* is a tuning parameter, where $q=2k$ is recommended in [12], though larger values provide higher numerical accuracy. To be precise, in [12] the authors provide a bound on the accuracy of the SVD returned by Algorithm 1 for $2 \le k \le 0.5\min (m,n)$, given by

7 $$\begin{aligned} \mathbb {E}\| X - U \Sigma V^\top \| \le \left[ 1 + 4 \sqrt{\frac{2 \min (m,n)}{k-1}} \right] ^{1/(2q+1)} \sigma _{k+1}, \end{aligned}$$

where $\mathbb {E}$ is the expectation with respect to the random Gaussian matrix, and $\sigma _{k+1}$ is the $(k+1)$th singular value of *X*. Note that the algorithm of [12] does not require a normalization of the eigenvectors as done in, for instance, the power method [29].

Our framework is based on the observation that all arithmetic operations in Algorithm 1 can be reformulated for input matrices of the type of Eqs. (3) and (4) such that they preserve sparse matrix algebra. Without loss of generality, we consider Eq. (4) in the following. An adapted algorithm to compute the eigenvectors of matrices in the form of Eq. (4) is given in Algorithm 2. The input of Algorithm 2 is the matrix $X = a \cdot v_2 \odot _c (G \ominus _c w_2)$ for which one wishes to compute the eigenvectors of $X^\top X$, the number of desired eigenvectors $k \in \mathbb {N}$, as well as $q \in \mathbb {N}$.

**Algorithm 2:**
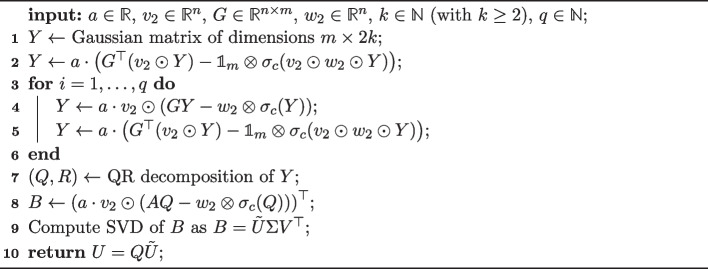
randomized fast SVD for $X^\top X$ with $X = a \cdot v_2 \odot _c (G \ominus _c w_2)$

In Algorithm 2, after initializing the matrix *Y* with Gaussian random numbers, the operation $Y \leftarrow (X X^\top )^q X Y$ needs to be performed. Importantly, as seen in lines 2–5, the structure of $X = a \cdot v_2 \odot _c (G \ominus _c w_2)$ allows one to separate the exponentiation into a part using sparse matrix algebra operations for *G* only, and simple outer products of lower dimension. Next, a QR decomposition is computed for *Y*, and $B \leftarrow Q^\top X$ is computed which again can be separated into a part using sparse matrix algebra only, and an outer product of lower dimensions. The resulting matrix *B* has the dimensions $(2k) \times n$ only, thus allowing for a fast SVD computation. Algorithm 2 returns $U=Q\tilde{U} \in \mathbb {R}^{m \times k}$ as done in Algorithm 1.

Note that the matrix *R* of the QR decomposition, as well as the matrices $\Sigma$ and $V^\top$ of the SVD are actually not needed if only the first *k* eigenvectors (given as columns in matrix *U*) are sought. Their computation can therefore be omitted. An adaptation of Algorithm 2 to Eq. (3) is straightforward and thus omitted in this article.

### Runtime considerations

As shown in [8], the effort to compute the four similarity matrices is $O(n m^2)$. If sparse algebra is used, the effort is $O(s n m^2)$, where $s \in [0,1]$ is the matrix sparsity parameter (the proportion of non-zero matrix entries). The resulting similarity matrix has dimensions $m \times m$ in all cases. A subsequent standard SVD to compute their eigenvectors has effort $O(m^3)$ [7]. Together, the effort for the computation of the similiary matrix and its eigenvectors via classic SVD is $O(n m^2 + m^3)$.

Using Algorithm 2, the computation of any of the four similarity matrices is entirely omitted. Running Algorithm 2 has a computational effort of $O(qk \cdot nm + k^2(n+m))$, thus making the computation of the first $k \in \mathbb {N}$ eigenvectors of any similarity matrix a linear time operation in both *m* and *n* (assuming the other is kept fixed).

The aforementioned runtime is derived as follows. Assume we apply the fast randomized SVD of Algorithm 1 to an input matrix $X \in \mathbb {R}^{n \times m}$ and *k* eigenvectors, using a fixed choice of *q*. In step 1, we generate $\Omega \in \mathbb {R}^{m \times (2k)}$ which requires effort *O*(*mk*) for writing all matrix entries. In step 2, we compute $X\Omega \in \mathbb {R}^{n \times (2k)}$, which requires effort *O*(*mnk*). Then, $X\Omega \in \mathbb {R}^{n \times (2k)}$ is left-multiplied with $X^\top \in \mathbb {R}^{m \times n}$ (likewise effort *O*(*mnk*)) and again left-multiplied with $X \in \mathbb {R}^{n \times m}$. This is done *q* times, leading to an effort of *O*(*qmnk*). The resulting matrix *Y* has dimensions $n \times (2k)$. In step 3, a QR decomposition is computed of $Y \in \mathbb {R}^{n \times (2k)}$. The *Q*-matrix of the QR-decomposition of *Y* has the same dimensions $n \times (2k)$, and since $n > 2k$, its computation typically costs $O(k^2n)$. In step 4, we compute *B* as $Q^\top X \in \mathbb {R}^{(2k) \times m}$, which requires effort *O*(*mnk*). In step 5, the SVD of $B \in \mathbb {R}^{(2k) \times m}$ costs $O(k^2\,m)$ since $m > 2k$. Note that actually, only a truncated SVD for the largest *k* singular values is needed, causing the matrix $\tilde{U}$ to have dimensions $(2k) \times k$. In step 6, the algorithm returns $U = Q\tilde{U} \in \mathbb {R}^{n \times k}$, which is computed as a matrix-matrix product in $O(nk^2)$. Together, we see that the effort of Algorithm 1 (and equivalently, of Algorithm 2) can be expressed as $O(qk \cdot nm + k^2(n+m))$.

## Experimental results

This section first presents experimental results on the numerical quality of the eigenvectors returned by Algorithm 2 when applied to the genomic relationship matrix (GRM) and the proposed approximate Jaccard matrix (section “Application to the 1000 Genomes Project data”). Afterwards, we experimentally verify both the numerical accuracy of Algorithm 2 (section “Investigation of numerical accuracy”), as well as the theoretical bounds on the accuracy of the Jaccard approximation (section “Verification of the theoretical bounds for the approximate Jaccard matrix”). We conclude with an experimental verification of the computational runtime of Algorithm 2 in section “Verification of theoretical runtimes”.

Throughout this section, we refer to five different algorithms to compute eigenvectors. Those are (1) a full traditional computation of eigenvectors with the function *eigen* in R, denoted as *traditional SVD*; (2) a truncated SVD computed with the function *eigs* of the R-package *RSpectra* [20], denoted as *truncated SVD*; (3) the *power method* with a fixed number of 100 iterations [29], which is a specialized algorithm to compute the first eigenvector only, denoted as *power method*; (4) the randomized SVD algorithm of [12] applied to some suitable matrix *X*, thereby computing the eigenvectors of $X^\top X$, denoted as *randomized SVD*; (5) the proposed Algorithm 2, denoted as Algorithm 2.

Since we aim to extract the eigenvectors of similarity matrices in this section, we note that the first three algorithms require the full calculation of the similarity matrix before extracting its eigenvectors. The randomized SVD and Algorithm 2 do not require the calculation of the similarity matrix and work on matrix *G* instead (see section “Fast computation of eigenvectors”).

### Application to the 1000 Genomes Project data

We apply Algorithm 2 to chromosome 1 of the 1000 Genomes Project [28], with the aim to visually examine the accuracy of the first two eigenvectors in population stratification figures.

We first prepare the raw data of the 1000 Genome Project using PLINK2 [26] using a cutoff value 0.01 for the option—*max-maf* to select rare variants. Moreover, we employ LD pruning with parameters—*indep-pairwise 2000 10 0.01*. All results focus on the European super population, containing 503 subjects and approximately 5 million rare variants.

**Fig. 1:**
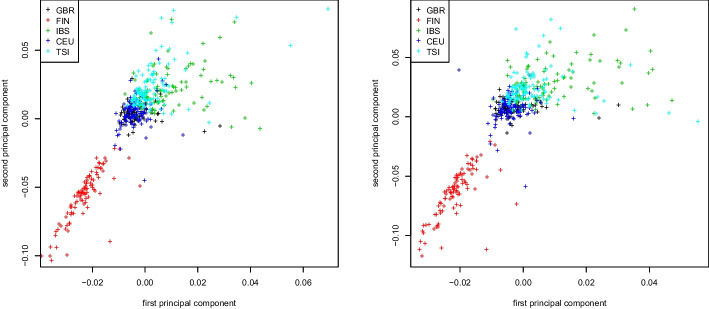
Genomic relationship matrix (GRM). First two principal components colored by population for the 1000 Genomes Project dataset. Truncated SVD (left) and Algorithm 2 (right)

Figure 1 shows results for the first two eigenvectors of the genomic relationship matrix (GRM), colored by subpopulation (GBR, FIN, IBS, CEU, TSI). The eigenvectors are computed with two methods: (1) we fully construct the GRM matrix on the 1000 Genomes Project dataset before extracting its eigenvectors using a truncated SVD; and (2) we use our proposed Algorithm 2, thereby avoiding the actual computation of the GRM matrix. We observe that the plots are almost identical. This is to be expected, as the GRM matrix allows for an exact decomposition in the from $X^\top X$ suitable for Algorithm 2, see section “Decomposition of three similarity matrices”.

**Fig. 2:**
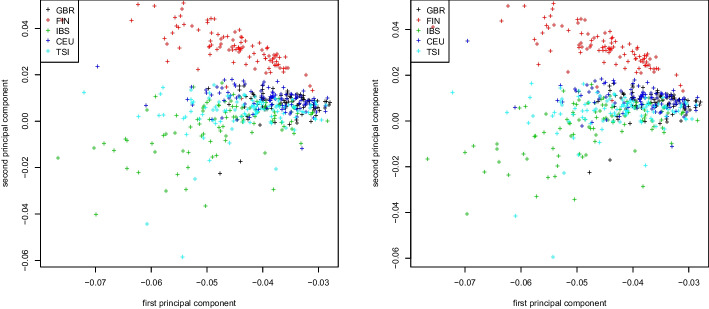
Covariance matrix. First two principal components colored by population for the 1000 Genome Project dataset. Truncated SVD (left) and Algorithm 2 (right)

**Fig. 3:**
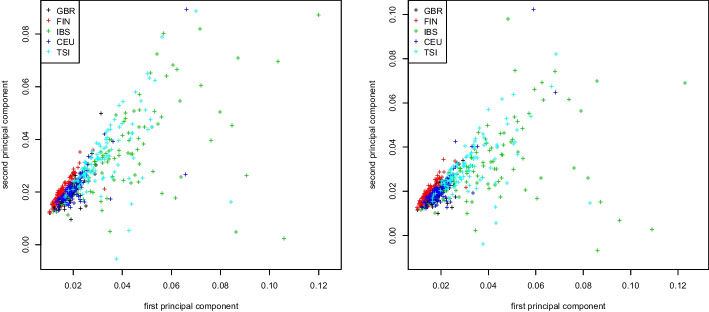
Weighted Jaccard matrix. First two principal components colored by population for the 1000 Genome Project dataset. Truncated SVD (left) and Algorithm 2 (right)

Similar plots for the covariance matrix and the weighted Jaccard matrix applied to the same dataset can be found in Figs. 2 and 3, respectively. As seen in both figures, the population stratification plots obtained by either computing the similarity matrix first and then its eigenvectors via truncated SVD, or by running Algorithm 2 are virtually identical. As before, this is to be expected as the decomposition into the form $X^\top X$ is exact for both the GRM and the weighted Jaccard matrix (see section “Decomposition of three similarity matrices”).

**Fig. 4:**
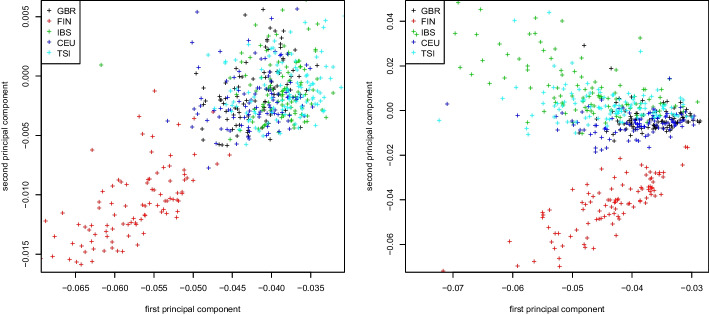
Jaccard matrix. First two principal components colored by population for the 1000 Genomes Project dataset. Truncated SVD (left) and Algorithm 2 (right)

We repeat this computation for the Jaccard matrix. Similarly to the previous case, we compute the first two eigenvectors by fully constructing the Jaccard matrix and extracting its eigenvectors using a truncated SVD, and by using the Jaccard approximation of section “A new approximation of the Jaccard similarity matrix” in connection with Algorithm 2. Figure 4 shows the first two eigenvectors of the original Jaccard and the approximate Jaccard matrix. We observe that here, the stratification plots are visibly different, though the approximate Jaccard matrix provides a very good stratification of the 1000 Genomes Project dataset.

### Investigation of numerical accuracy

The principal components of the genomic relationship matrix in Fig. 1, computed once with a truncated SVD and once with Algorithm 2, are almost identical. However, although the decomposition in Eqs. (3) and (4) is exact for the genomic relationship matrix, the two plots in Fig. 1 exhibit small differences attributed to numerical error/ approximations. To investigate those, we measure the $L_2$ distance and sine of angle between the two first principle components.

For the experiments in this and all following subsections, we employ simulated data. We create sparse matrices *G* of dimensions $n \times m$, where a proportion $\pi \in [0,1]$ of entries is set to one (acting as nonzero alleles). The dimensions $n \times m$ are given individually for each experiment. Unless stated otherwise, we set $\pi =0.1$.

**Fig. 5:**
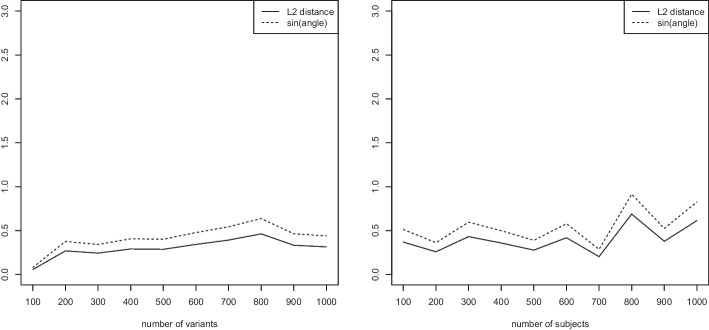
Measured $L_2$ distance and sine of angle between the first eigenvector of the genomic relationship matrix (GRM) computed with both a truncated SVD and with Algorithm 2. Variable number of variants *n* while keeping $m=100$ fixed (left) and variable number of subjects *m* while keeping $n=100$ fixed (right)

Figure 5 shows the scaling of the numerical error as a function of the number of variants *n* while keeping $m=100$ fixed, and as a function of the number of subjects *m* while keeping $n=100$ fixed. We observe that the error does not seem to grow with a scaling of the input, but rather plateaus at a low level.

### Verification of the theoretical bounds for the approximate Jaccard matrix

We are interested in quantifying further the numerical tradeoff made when computing the eigenvectors of the approximate Jaccard matrix. Additionally, we aim to verify the theoretical bounds on the approximate eigenvectors derived in section “Theoretical error bounds on the eigenvectors of the Jaccard approximation”.

To this end, we again investigate both the validity and the scaling behavior of the bounds of section “Theoretical error bounds on the eigenvectors of the Jaccard approximation” as a function of the number of variants *n*, the number of subjects *m*, the proportion of nonzero alleles, and higher order eigenvectors. We use the simulation setting of section “Investigation of numerical accuracy”.

**Fig. 6:**
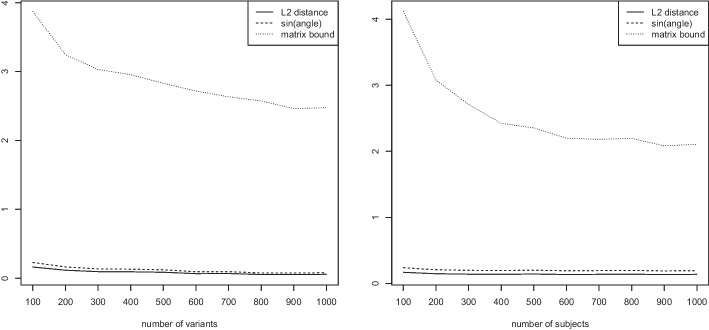
Measured $L_2$ distance between the first eigenvector of the Jaccard matrix and the approximate Jaccard matrix, angle bound of Eq. (5), and matrix bound of Eq. (6). Variable number of variants *n* while keeping $m=100$ fixed (left) and variable number of subjects *m* while keeping $n=100$ fixed (right)

Figure 6 (left) shows results as the number of variants *n* increases while keeping the number of subjects $m=100$ fixed. The figure displays the measured $L_2$ distance between the first eigenvector of the Jaccard matrix and the approximate Jaccard matrix, the angle bound of Eq. (5), and the matrix bound of Eq. (6). Similarly, in Fig. 6 (right) we vary the number of subjects *m* while keeping the number of variants $n=100$ fixed. In both cases, we observe that the measured $L_2$ distance between the first eigenvector of the Jaccard matrix and its approximation is negligible. The angle bound (which requires the computation of both eigenvectors) seems to be a very tight bound, while the matrix bound (which requires no computation of eigenvectors and is thus an a priori bound) is valid but less tight. This is to be expected, as less information is required to compute the matrix bound. In particular, in can be computed without having computed any eigenvectors.

**Fig. 7:**
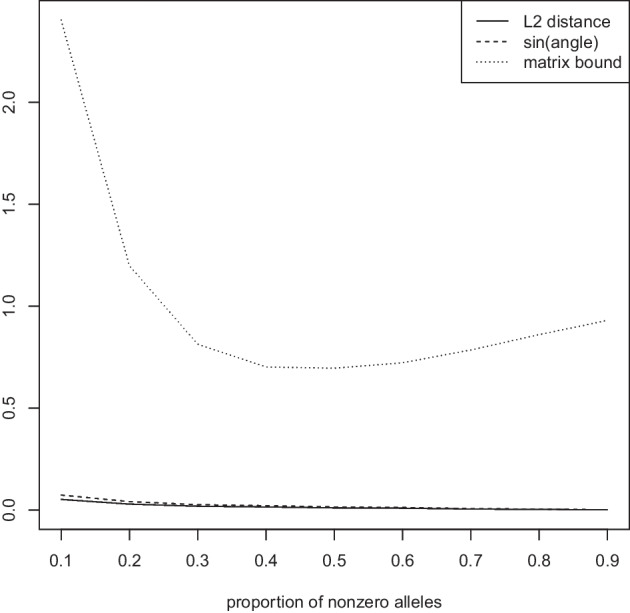
Measured $L_2$ distance between the first eigenvector of the Jaccard matrix and the approximate Jaccard matrix, angle bound of Eq. (5), and matrix bound of Eq. (6). Proportion $\pi \in \{0.1,\ldots ,0.9\}$ of entries 1 while keeping the number of variants $n=1000$ and the number of subjects $m=100$ fixed

Figure 7 investigates the accuracy of the theoretical bounds as a function of the proportion $\pi \in \{0.1,\ldots ,0.9\}$ determining the sparseness of the matrix *G* while keeping the number of variants $n=1000$ and the number of subjects $m=100$ fixed. We observe that again, the error in $L_2$ distance between the eigenvector computed for the Jaccard and approximate Jaccard matrices is negligible. The angle bound is again tight. The matrix bound is valid, more relaxed than the angle bound, and exhibits its closest bound for around $\pi = 0.4$.

**Fig. 8:**
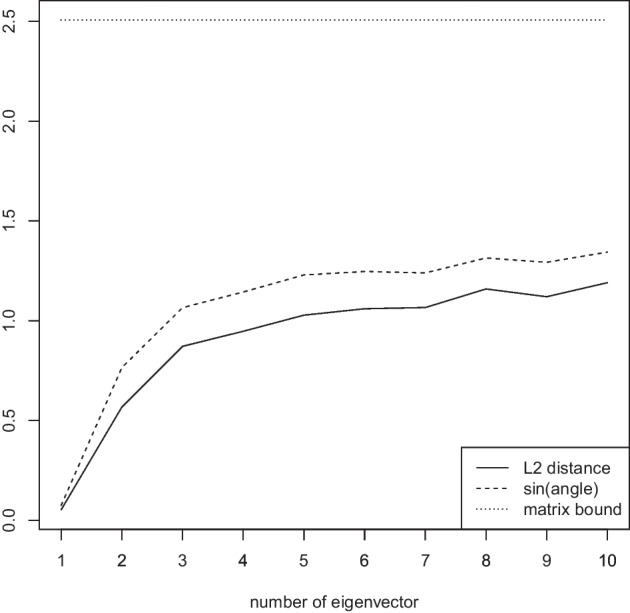
Measured $L_2$ distance between the first eigenvector of the Jaccard matrix and the approximate Jaccard matrix, angle bound of Eq. (5), and matrix bound of Eq. (6). Bound progression for the first 10 eigenvectors while keeping the number of variants $n=1000$, the number of subjects $m=100$, and $\pi =0.1$ fixed

Finally, Fig. 8 applies the bounds of Eqs. (5) and (6) to higher order eigenvectors. We observe that the approximate Jaccard matrix is more accurate for the first eigenvectors than the later ones. The angle bound nicely follows the actual observed $L_2$ norm between the eigenvectors computed for the Jaccard matrix and its approximation, while the matrix bound is the same for all since it only takes the Jaccard matrix and its approximation into account (but no information on the eigenvector being computed).

### Verification of theoretical runtimes

Finally, we aim to investigate the empirical runtime scalings of the algorithms discussed in the previous sections, in particular we aim to verify the theoretical runtimes derived in section “Runtime considerations”.

As shown in section “Runtime considerations”, the calculation of a full similarity measure with a subsequent eigenvector computation using a (truncated) SVD has a theoretical runtime of $O(n m^2 + m^3)$, while Algorithm 2 has a theoretical runtime of $O(qk \cdot nm + k^2(n+m))$.

We consider two scenarios. The first scenario examines the computation of the leading eigenvector only, in which case it is sensible to use a specialized algorithm such as the power method [29]. The second scenario examines the general case of $k>1$ eigenvectors being computed. Each of the two scenarios is yet again considered separately for dense and sparse matrices. For sparse matrix support, we employ the R-package *Matrix* on CRAN [2]. We select the genomic relationship matrix as the similarity measure in our runtime comparisons.

**Fig. 9:**
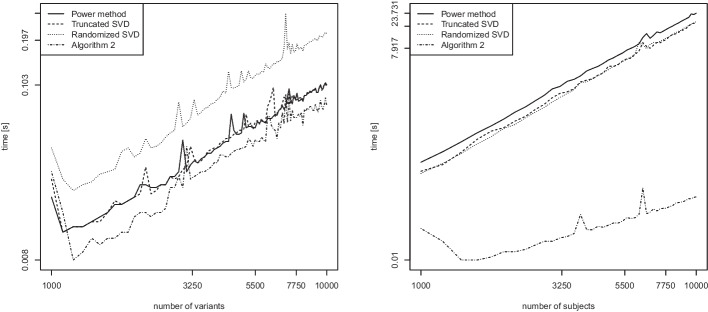
Leading eigenvector of the genomic relationship matrix (GRM) for dense matrices. Runtime (in seconds) as a function of the number of variants *n* while keeping $m=100$ fixed (left), and as a function of the number of subjects *m* while keeping $n=100$ fixed (right). Log scale on both axes

We first consider the computation of the leading eigenvector for dense matrices. We compare the power method, the truncated SVD, the randomized SVD (Algorithm 1), and our proposed Algorithm 2. Figure 9 shows empirical runtime scalings in the number of variants *n* (left) and the number of subjects *m* (right) while keeping the other fixed at value 100.

In Fig. 9 (left) investigating the scaling in *n*, all lines have a slope of around 1. This is in good accordance with the theoretical runtime which predicts a linear scaling in *n* for all methods. In Fig. 9 (right) investigating the scaling in *m*, the lines have slopes of around 2 for the power method, truncated SVD, and randomized SVD. The slope for Algorithm 2 is 0.87. The experimental runtime dependence for the power method and the truncated SVD are therefore as expected since these methods rely on the computation of the GRM matrix first, which requires quadratic effort in *m* [8]. The runtime dependence of the randomized SVD turns out to be higher than expected. The dependence of Algorithm 2 is roughly linear and thus matches its theoretical runtime.

**Fig. 10:**
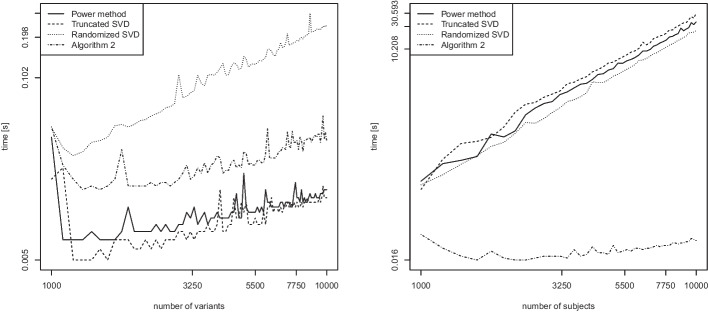
Leading eigenvector of the genomic relationship matrix (GRM) for sparse matrices. Runtime (in seconds) as a function of the number of variants *n* while keeping $m=100$ fixed (left), and as a function of the number of subjects *m* while keeping $n=100$ fixed (right). Log scale on both axes

We repeat the same experiment in sparse matrix algebra. Figure 10 displays results for the computation of the leading eigenvector of sparse matrices. Due to the usage of fast sparse matrix algebra in R, we observe in Fig. 10 (left) that all methods attain an empirical runtime that is either roughly linear (the slope for the randomized SVD is 0.96) or even faster than linear (empirical slopes of roughly 0.5) in the number of variants *n*. When looking at the scaling in the number of subjects *m*, Fig. 10 (right) shows that the power method, truncated SVD, and randomized SVD all achieve a similar runtime scaling with slopes of around 2 (as predicted by the theoretical runtime). The runtime scaling of Algorithm 2 is roughly linear and thus more favorable.

**Fig. 11:**
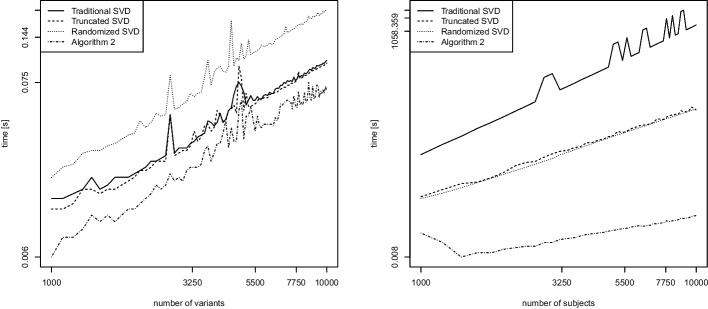
Computation of $k=2$ eigenvectors of the GRM matrix for dense matrices. Runtime (in seconds) as a function of the number of variants *n* while keeping $m=100$ fixed (left), and as a function of the number of subjects *m* while keeping $n=100$ fixed (right). Log scale on both axes

We now consider the second scenario in which we compute $k=2$ eigenvectors. In contrast to the previous comparisons, we include the traditional (full) eigenvector computation. As before, the other algorithms included in the comparison are the truncated SVD, the randomized SVD (Algorithm 1), and our proposed Algorithm 2. Figure 11 shows empirical runtime scalings for computing $k=2$ eigenvectors in dense matrix algebra, again in the number of variants *n* (left) or the number of subjects *m* (right) while keeping the other fixed at value 100.

In Fig. 11 (left) investigating the scaling in *n*, all lines again have a slope of around 1, which is in good accordance with the theoretical linear runtime. In Fig. 11 (right) investigating the scaling in *m*, we observe three different slopes. The traditional SVD has an empirical slope of 3.10, thus hinting at a cubic runtime, as expected. The truncated SVD and the randomized SVD have slopes of roughly 2.05 and 2.10, as seen previously. The slope for Algorithm 2 is around 0.86 and thus roughly matches its predicted linear theoretical runtime.

**Fig. 12:**
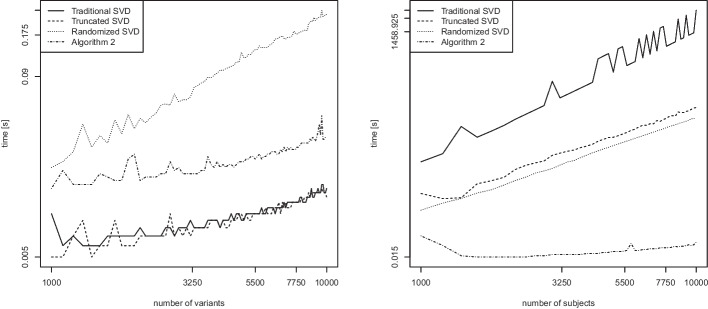
Computation of $k=2$ eigenvectors of the GRM matrix for sparse matrices. Runtime (in seconds) as a function of the number of variants *n* while keeping $m=100$ fixed (left), and as a function of the number of subjects *m* while keeping $n=100$ fixed (right). Log scale on both axes

The corresponding results for computing $k=2$ eigenvectors in sparse matrix algebra are displayed in Fig. 12. As shown in Fig. 12 (left), the runtime scaling of the randomized SVD is roughly linear (empirical slope of roughly 1.1), while the runtimes of the other three methods are even faster than linear (empirical slopes of roughly 0.5), an observation that is attributed to the fast sparse matrix algebra in R. This result matches the one observed for the computation of the leading eigenvector of sparse matrices in Fig. 10 (left).

Figure 12 (right) displays the asymptotic scalings for computing $k=2$ eigenvectors in sparse matrix algebra. As it seems, the traditional SVD does not make good use of the sparse matrix algebra and attains an empirical cubic slope (see the left panel of Fig. 11), while the truncated SVD and the randomized SVD have slopes of roughly 2. The slope for Algorithm 2 in connection with sparse matrix algebra is only around 0.2 and thus below its predicted linear theoretical runtime.

Overall, these results indicate that Algorithm 2 is faster both experimentally and theoretically than many of the traditional approaches for computing the leading or the first leading eigenvectors, both in dense matrix algebra (Figs. 9 and 11) and in sparse matrix algebra (Figs. 10 and 12).

## Discussion

This article considered the fast and efficient computation of eigenvectors of four similarity matrices, the covariance matrix, the Jaccard matrix, the weighted Jaccard matrix, and the genomic relationship matrix. The computation of such eigenvectors is a standard tool in computational genetics, and it is of importance for correcting linear regressions, revealing population stratification, and many more application areas.

In this contribution, we first introduce a unified way to express the covariance matrix, the weighted Jaccard matrix, and the genomic relationship matrix which allows one to efficiently compute their eigenvectors in sparse matrix algebra using an adaptation of a fast SVD algorithm of [12]. Notably, the only requirement for the proposed Algorithm 2 to work efficiently is the existence of efficient row-wise and column-wise subtraction and multiplication operations of a vector with a sparse matrix. Those are standard operations commonly available in sparse matrix packages. An exception is the Jaccard matrix, which does not have a structure applicable for fast SVD computations. Second, we thus introduce a new approximate Jaccard matrix to which the fast SVD computation is applicable. Third, we establish guaranteed theoretical bounds on the distance (in $L_2$ norm and angle) between the principal components of the Jaccard matrix and the ones of our proposed approximation. These a priori bounds allow the user to gauge in advance the trade-off between obtained speedup and sacrificed accuracy when using the proposed approximate Jaccard matrix.

We verify theoretically and experimentally that our proposed Algorithm 2 keeps the predicted error bounds and has a more favorable runtime scaling than the traditional computation of a similarity measure and a subsequent extraction of principal components.

The derivation of further theoretical properties of the proposed approximate Jaccard matrix is left for future work.

## Data Availability

The datasets analyzed during the current study are available from the 1000 Genome Project under the identifier *EUR Phase3 v5 hg38/GRCh38*, see https://www.internationalgenome.org/.
